# Differentiating severe and non-severe lower respiratory tract illness in patients hospitalized with influenza: Development of the Influenza Disease Evaluation and Assessment of Severity (IDEAS) scale

**DOI:** 10.1371/journal.pone.0258482

**Published:** 2021-10-21

**Authors:** Eric J. Chow, Mark W. Tenforde, Melissa A. Rolfes, Benjamin Lee, Shreya Chodisetty, Julio A. Ramirez, Alicia M. Fry, Manish M. Patel

**Affiliations:** 1 Influenza Division, National Center for Immunization and Respiratory Diseases, Centers for Disease Control and Prevention, Atlanta, Georgia, United States of America; 2 Epidemic Intelligence Service, Center for Surveillance, Epidemiology and Laboratory Services, Centers for Disease Control and Prevention, Atlanta, Georgia, United States of America; 3 Division of Infectious Diseases, Department of Medicine, University of Louisville School of Medicine, Louisville, Kentucky, United States of America; Kaohsuing Medical University Hospital, TAIWAN

## Abstract

**Background:**

Experimental studies have shown that vaccination can reduce viral replication to attenuate progression of influenza-associated lower respiratory tract illness (LRTI). However, clinical studies are conflicting, possibly due to use of non-specific outcomes reflecting a mix of large and small airway LRTI lacking specificity for acute lung or organ injury.

**Methods:**

We developed a global ordinal scale to differentiate large and small airway LRTI in hospitalized adults with influenza using physiologic features and interventions (PFIs): vital signs, laboratory and radiographic findings, and clinical interventions. We reviewed the literature to identify common PFIs across 9 existing scales of pneumonia and sepsis severity. To characterize patients using this scale, we applied the scale to an antiviral clinical trial dataset where these PFIs were measured through routine clinical care in adults hospitalized with influenza-associated LRTI during the 2010–2013 seasons.

**Results:**

We evaluated 12 clinical parameters among 1020 adults; 210 (21%) had laboratory-confirmed influenza, with a median severity score of 4.5 (interquartile range, 2–8). Among influenza cases, median age was 63 years, 20% were hospitalized in the prior 90 days, 50% had chronic obstructive pulmonary disease, and 22% had congestive heart failure. Primary influencers of higher score included pulmonary infiltrates on imaging (48.1%), heart rate ≥110 beats/minute (41.4%), oxygen saturation <93% (47.6%) and respiratory rate >24 breaths/minute (21.0%). Key PFIs distinguishing patients with severity < or ≥8 (upper quartile) included infiltrates (27.1% vs 90.0%), temperature ≥ 39.1°C or <36.0°C (7.1% vs 27.1%), respiratory rate >24 breaths/minute (7.9% vs 47.1%), heart rate ≥110 beats/minute (29.3% vs 65.7%), oxygen saturation <90% (14.3% vs 31.4%), white blood cell count >15,000 (5.0% vs 27.2%), and need for invasive or non-invasive mechanical ventilation (2.1% vs 15.7%).

**Conclusion:**

We developed a scale in adults hospitalized with influenza-associated LRTI demonstrating a broad distribution of physiologic severity which may be useful for future studies evaluating the disease attenuating effects of influenza vaccination or other therapeutics.

## Introduction

Influenza virus infection can range in clinical severity from asymptomatic to severe symptomatic illness that might be fatal [1–6]. Vaccination confers partial protection against medically attended illness from any influenza virus infection, estimated from 22%-64%, and varies from year to year [7]. As breakthrough infections may occur after vaccination, it is important to also understand if vaccination reduces the severity of illness (i.e., attenuates disease) which may help promote the public health benefits of annual vaccination [8–10]. For childhood vaccines that provide partial protection in a population, protection might be higher against severe disease as compared with milder illness, which could influence vaccination policies and decisions [11–15]. For example, on the promise of attenuating disease severity, second-generation rotavirus vaccines have been successfully deployed worldwide resulting in marked reductions in rotavirus deaths and hospitalizations [12, 16, 17].

Characterization of disease attenuating effects of vaccination in real-world conditions requires two important considerations. First, a standardized definition of acute illness severity is necessary. Second, this severity definition must be specific for the acute pathology that immunity is expected to attenuate. The pathology of influenza-associated lower respiratory tract infection (LRTI) can be varied and include immunopathology from pro-inflammatory immune responses, direct virus-mediated damage, or inflammation related to bacterial superinfection [3, 18–21]. Triggers for the immunopathology include tropism of the influenza virus (seasonal, pandemic vs novel influenza viruses), virus dose, site of inoculum (upper vs lower airway), and host defense mechanisms or genetic susceptibility [3, 22–29]. Disease may be confined to the large airway, causing transient tracheobronchitis (herein referred to as non-severe LRTI) [3, 6]. However, viral damage may also extend to small airways, causing acute lung injury, primary viral pneumonia, or secondary bacterial infection, with or without systemic extrapulmonary involvement (herein referred as severe LRTI) [4, 5, 18]. In the absence of pre-existing mucosal immunity, breakthrough infections can occur but recall of immune responses after infection might attenuate influenza disease by limiting viral replication and spread to the small airway or extrapulmonary organ systems [27, 30–41].

While experimental virus challenge studies in animals and humans have shown the attenuating effects of influenza vaccination [27, 30–41], evidence from clinical studies is conflicting [10, 42–44]. The discrepancy might be due to clinical studies relying on non-specific outcomes such as influenza-associated hospitalization or intensive care unit admission (ICU) as surrogates for disease progression that vaccination is expected to attenuate. However, clinical outcomes are influenced not only by severity of LRTI but also by factors such as age, underlying conditions, and healthcare seeking and treatment practices. For example, while influenza tracheobronchitis in healthy hosts often results in an uncomplicated clinical course, infection in patients with compromised reserve (e.g., chronic obstructive pulmonary disease [COPD] or frailty) can trigger complicated outcomes such as hospitalization, ICU admission, or death [44]. In turn, patients hospitalized with LRTI can be a mix of complicated large airway LRTI and small airway LRTI. To the extent that immunity confines infection to the large airways, clinically differentiating disease pathology is important for evaluating vaccine-mediated disease attenuation. Quantifying severity using a “global” ordinal scale inclusive of clinical features, clinical interventions, laboratory values, and imaging findings that correlate with pathology can improve the specificity of disease outcomes that vaccination can attenuate and reproducibility of studies evaluating influenza vaccine effectiveness against severe disease [13, 14]. Such a global scale grades disease severity based on pre-specified parameters assumed to be associated with acute infection and cannot be validated like traditional severity scores designed to “predict” poor clinical outcomes. The U.S. Food and Drug Administration recognizes that in absence of a single, gold standard endpoint for seriously ill hospitalized influenza patients, clinical trials should consider clinical severity endpoints on an ordinal scale [45]. An expert Working Group on influenza therapeutic trials proposed the development of such an ordinal scale for facilitating the evaluation of efficacy of antivirals among hospitalized patients and the World Health Organization adopted a similar approach for evaluating COVID-19 vaccines [46, 47]. To our knowledge, such an ordinal scale does not exist for evaluation of influenza vaccine-mediated disease attenuation.

Our hypothesis is that illness severity relates to viral inoculum and replication, and that viral spread results in hyperinflammation and severe LRTI [2, 4–6, 22, 23, 27, 33, 48]. Our study aims were: 1) to identify quantitative clinical features that might differentiate non-severe and severe LRTI among hospitalized patients; 2) to develop a scale to grade influenza illness using these clinical features; and 3) to apply and evaluate the scale to categorize and compare the severity of influenza in patients in a clinical trial dataset.

## Methods

### Physiological parameters and clinical interventions correlating with critical illness

As compared with non-severe LRTI requiring hospitalization, we hypothesized that patients with severe LRTI would be more likely to manifest derangements in physiological parameters from normal values and require intensive clinical interventions. We took a systematic approach to identify quantitative physiological features and interventions (PFI) associated with severity of acute respiratory illness or sepsis. We focused on a parsimonious set of PFIs that 1) were consistently identified across various contemporary severity scores; 2) were practical to collect among hospitalized patients; and 3) allowed grading of a spectrum of LRTI [3].

We first performed a qualitative search of the literature to identify commonly used contemporary severity scores associated with critical outcomes in patients with influenza and non-influenza acute respiratory illness and sepsis. We searched PubMed MEDLINE between January 2000 –December 2020 for “influenza” OR “community-acquired pneumonia” OR “sepsis” AND “severity” AND “scale” OR “score” OR “model” OR “prediction.” We excluded severity scores related to COVID-19 due to absence of co-circulation with influenza and the unique presentation of severe COVID-19 compared with influenza [49]. We included all-cause pneumonia and sepsis because severity scores confined to laboratory-confirmed influenza are scarce and because of the likely overlaps in the physiological features of clinical deterioration in these conditions [50–52]. We also surmised that physiological features for these conditions would capture acute extrapulmonary manifestations of influenza virus infection such as cardiac injury, encephalopathy, and other organ dysfunctions [4].

From a review of abstracts, we identified contemporary studies that developed and validated scores or identified physiological parameters predicting critical illness. We reviewed the reference lists of all included articles and reviews to identify key additional sources of data, particularly older versions of updated severity scores. From this search, we identified nine severity scores or studies that defined physiological parameters associated with critical illness (**Table 1**) [53–61].

**Table 1:** Published severity scores^a^ informing the physiologic features and interventions for the Influenza Disease Evaluation and Assessment of Severity (IDEAS) scale.

Variable Name	Severity Index or Study
Temperature	APACHE II
	National Early Warning Score
	Pneumonia Severity Index
	Simple Clinical Score
	Simplified Acute Physiology Score II
Respiratory Rate	APACHE II
	CURB-65
	National Early Warning Score
	Pneumonia Severity Index
	SMART-COP
	qSOFA
	Simple Clinical Score
Heart Rate	APACHE II
	National Early Warning Score
	Pneumonia Severity Index
	SMART-COP
	Simple Clinical Score
	Simplified Acute Physiology Score II
Mean Arterial Pressure	APACHE II
	SOFA
Oxygen Saturation	National Early Warning Score
	SOFA^b^
	SMART-COP
	Simple Clinical Score
White Blood Cell Count	APACHE II
	Simplified Acute Physiology Score II
Serum Sodium	APACHE II
	Pneumonia Severity Index
	Simplified Acute Physiology Score II
Arterial Blood Gas, pH	APACHE II
	Pneumonia Severity Index
	SMART-COP
Altered Mental Status	National Early Warning Score
	Pneumonia Severity Index
	qSOFA
	Simple Clinical Score
	CURB-65
Infiltrate on Imaging	SMART-COP
Vasopressor Use	SOFA
Mechanical Ventilation	SOFA

Abbreviations: APACHE II = Acute Physiologic Assessment and Chronic Health Evaluation II; CURB-65 = Confusion, Urea > 7 mmol/l, Respiratory rate ≥ 30/min, and low Blood pressure (diastolic blood pressure ≤ 60 mm Hg or systolic blood pressure < 90 mm Hg, age ≥ 65 years; SMART-COP = Systolic blood pressure, Multilobar chest radiography involvement, Albumin level, Respiratory rate, Tachycardia, Confusion, Oxygenation, and arterial pH; qSOFA = Quick Sequential Organ Failure Assessment; SOFA = Sequential Organ Failure Assessment.

^a^ References in the primary manuscript: APACHE II [56], National Early Warning Score [53], Pneumonia Severity Index [55], Simplified Clinical Score [60], Simplified Acute Physiology Score II [57], CURB-65 [58], SMART-COP [61], qSOFA [59], SOFA [54].

^b^ Note that the SOFA score uses partial pressure of oxygen (PaO2) / fraction of inspired oxygen (FiO2) ratio.

### Developing the Influenza Disease Evaluation and Assessment of Severity (IDEAS) scale

To overcome the challenges of using outcome-based scales that were not designed to evaluate vaccination-mediated attenuation, we creatively developed the Influenza Disease Evaluation and Assessment of Severity (IDEAS) scale based on PFIs. First, two clinicians (EC and MP) reviewed severity scores and predictors of illness severity and used clinical judgment to discern physiological features of acute illness severity that were common across the scores and studies, emphasizing parameters that are typically available in hospitalized patients (**Table 1)**. We categorized PFIs into four groups: vital signs (temperature, respiratory rate, heart rate, mean arterial pressure, pulse oximetric or oxygen saturation); laboratory findings (white blood cell count, serum sodium, arterial blood gas pH); clinical findings (pulmonary infiltrate on imaging; acute mental status changes); and clinical interventions (vasopressor use and mechanical ventilation) (**Table 2**). Discrepancies in characterization were adjudicated through consensus.

**Table 2:** Point Allocation based on Physiologic Features and Interventions (PFI) for the Influenza Disease Evaluation and Assessment of Severity (IDEAS) scale.

				Subtracting Points
Category	Severity Variables	Value Ranges	Point Allocation	-2 from the total score for age >70
Vital Signs	Temperature	≥ 39.1	3	
		38.1–39.0	2	
		36.1–38.0	0	
		35.1–36.0	2	
		< 35.1	3	
	Respiratory Rate, breaths/min	≥25	3	-1 if patient has history of CLD^a^ or CHF
		<25	0	
	Heart Rate, beats/min	>130	3	
		110–130	2	
		60–109	0	
		41–59	1	
		≤40	3	
	Mean Arterial Pressure, mmHg	< 60	3	
		≥60	0	
	Oxygen Saturation, %	> 93%	0	-2 if patient has history of CLD or CHF
		90–93%	2	
		<90%	3	
Laboratory Findings	White Blood Cell Count, cells x10^3^/mm^3^	≥40	3	
		20–39	2	
		15–19	1	
		3–14	0	
		1–2	1	
		<1	2	
	Serum Sodium, mEq/L,	<120	3	-1 if patient has history of CHF
		120–125	1	
		>125	0	
	Arterial Blood Gas, pH	≥7.6	3	
		7.5–7.59	1	
		7.33–7.49	0	
		7.25–7.32	1	
		<7.25	3	
Mental Status	Altered Mental Status	Altered	2	
		Not Altered	0	
In-Hospital Interventions	Infiltrate on Imaging	Yes	5	
		No	0	
	Vasopressor Use	Yes	1	
		No	0	
	Mechanical Ventilation, days from symptom onset	Invasive 0–3	3	-1 if patient has history of CLD or CHF
		Invasive > 3	2	
		Noninvasive 0–3	2	
		Noninvasive >3	1	
		No mech vent	0	

Abbreviations: CHF = congestive heart failure; CLD = chronic lung disease.

^a^ CLD is defined as history of chronic obstructive pulmonary disease or baseline home oxygen use.

Second, we developed the modifiable IDEAS scale by allocating points to each of the PFI. Values of PFIs were considered during the first 24 hours of admission, except for vasopressor use, or mechanical ventilation. Use of SpO2/FiO2 (where SpO2 represents the oxygen saturation and FiO2 represents fractional inspired oxygen ratio) would be favorable to oxygen saturation alone [62], although FiO2 concentrations might not be recorded in all datasets. We modeled our approach after “track-and-trigger” systems that have been used in various settings globally to use physiological parameters to identify and treat patients with clinical deterioration [50–52, 63]. The magnitude of the scoring reflects the deviation of the parameter from the norm. The individual scores of each PFI would be aggregated to develop a global score for each patient. With the exception of infiltrate on imaging, each PFI was based on a 0–3 point scale (either continuous or dichotomous), using cutoffs established by scores from which the PFI was derived. We weighted three of the PFIs based on our understanding of influenza pathogenesis that considers evidence from experimental inoculation studies and clinical experience. We up-weighted presence of infiltrates on imaging to a score of 5 to account for lower lung injury, which we deem to be a primary manifestation of severe influenza illness that vaccination is expected to attenuate. We down-weighted two common features of hospitalized patients that might bias the score upward and promote misclassification of disease severity: 1) *Age* [64]: we subtracted 2 points from the global score for age >70 years to account for higher prevalence of baseline derangements that overlap with acute illness severity (e.g., dementia or baseline abnormities in sodium levels) and poor physiologic reserve that increases mortality which could lower the threshold for requiring interventions such as ventilation. 2) *Underlying conditions* [65, 66]: we subtracted points from PFI features that could be affected by presence of congestive heart failure (oxygen saturation, ventilation, serum sodium levels, or respiratory rate) and chronic lung disease (oxygen saturation, ventilation, or respiratory rate) commonly seen in adult patients hospitalized with acute respiratory illness.

### Applying the IDEAS scale

We applied the IDEAS scale to data from the Rapid Empiric Treatment with Oseltamivir Study (RETOS), a randomized clinical trial evaluating the effectiveness of oseltamivir treatment versus standard of care in adults (aged >18 years) hospitalized with LRTI in Louisville, Kentucky from 2010–2013 (clinicaltrials.gov, #NCT01248715) [67, 68]. In brief, patients were eligible for the study if they presented with clinician-diagnosed community-acquired pneumonia, acute exacerbation of COPD, or acute bronchitis and did not receive oseltamivir or zanamivir on hospital admission. All enrollees were systematically tested using a molecular assay (Luminex xTAG) for 12 respiratory viral pathogens by nasopharyngeal swab obtained by study staff. We included laboratory-confirmed influenza cases and patients who tested negative for influenza in the months between the first and last influenza case for each influenza season.

### Data analysis

Because our primary motivation for developing IDEAS was to evaluate vaccine attenuation of influenza disease, we focused primarily on laboratory-confirmed influenza cases. Grading of disease severity in test-negative controls is not typically relevant for vaccine attenuation analysis. We conducted a descriptive analysis of the study cohort, using count (percentage) and median (interquartile range [IQR]) to summarize proportions and continuous variables. We assessed the distribution of the subjects on the severity scale using histograms and box plots. We stratified patients by the results of influenza testing. We used the upper quartile of the severity score to define high vs those in the bottom three quartiles as low severity and compared patient characteristics of these two groups. We looked at the frequency of PFIs in the patients with high and low severity scores and considered a 20% difference in prevalence to be a meaningful distinguishing feature. Mean difference in scores were also compared by patient characteristics. In the RETOS dataset, we did not impute for missingness and conducted a sensitivity analysis by removing variables from the IDEAS scale with more than 30% missingness in the dataset. Only pH by arterial blood gas met that criteria in the RETOS dataset with missing data on 56% of patients. Testing for statistical significance was conducted using the Pearson chi-squared tests to compare categorical variables or Wilcoxon rank-sum test for medians to compare continuous data. Statistical significance was established as p value <0.05. Data analysis was performed using SAS Version 9.4 (Cary, North Carolina). This activity was determined to meet the definition of research [45 CFR 46.102(l)] involving human subjects [45 CFR 46.102 (e)(1)] and Institutional Review Board (IRB) approval was provided by the University of Louisville Human Subjects Research Protection Program Office (Protocol 10.0465) and the Robley Rex Veterans Administration Medical Center IRB (protocol 0068/00325). The Centers for Disease Control and Prevention IRB granted reliance on local ethical review approvals. Written informed consent was obtained on all participants.

## Results

Of the 1020 overall participants enrolled during the influenza seasons, 210 (20.5% had laboratory-confirmed influenza), the median age was 62 years (IQR, 52 to 72 years), 47.8% were female, 69.2% were overweight or obese, 80.6% were ever smokers (35.4% current smokers), and 28.2% were hospitalized in the prior 90 days before the index hospitalization. Underlying conditions were common: 70.4% had essential hypertension, 56.3% COPD, 37.3% diabetes mellitus, 29.1% coronary artery disease, and 26.6% had congestive heart failure.

### Physiologic Features and Interventions (PFIs)

**Fig 1:**
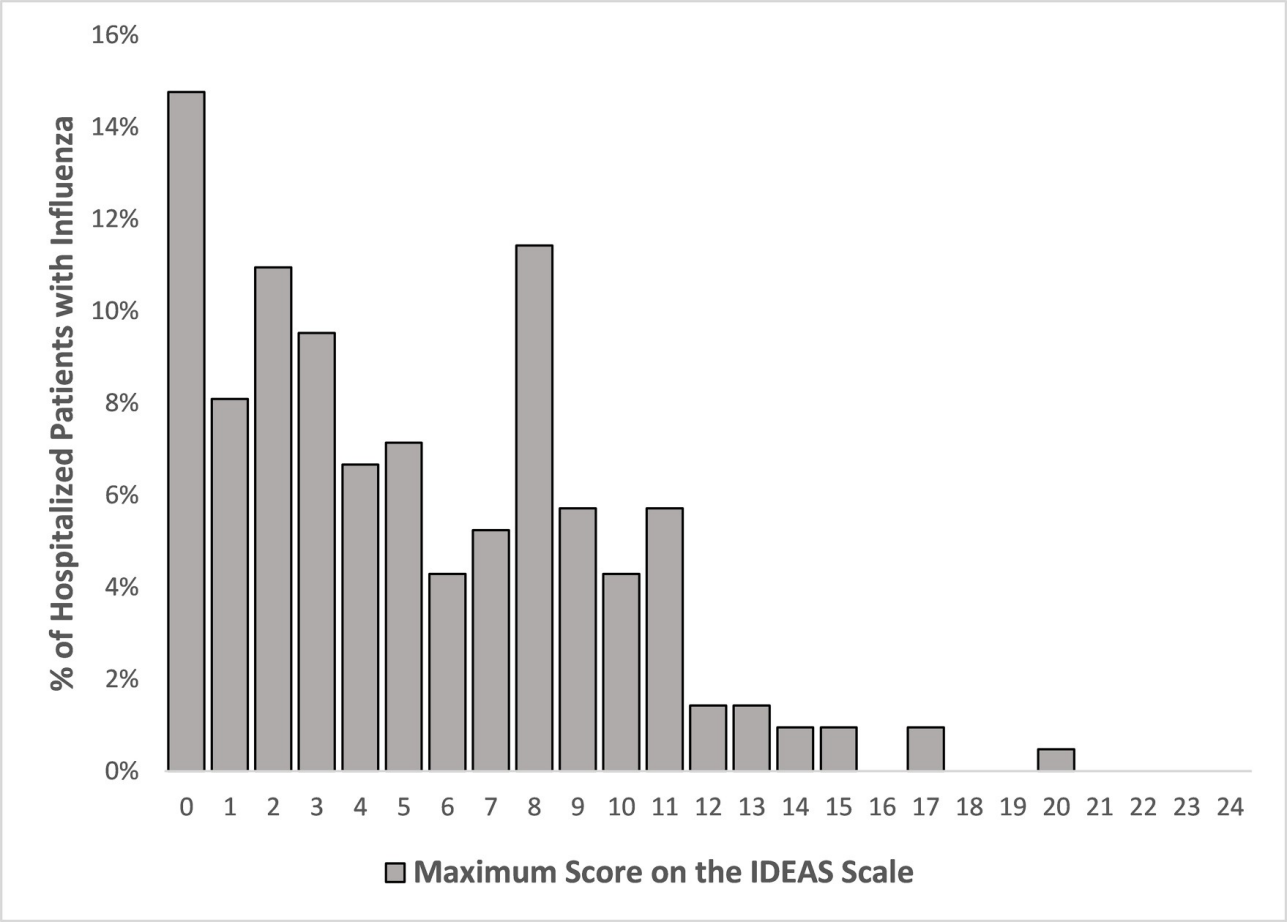
Distribution of hospitalized influenza-positive patients based on the maximum score on the Influenza Disease Evaluation and Assessment of Severity (IDEAS) scale.

With the exception of presence of infiltrates on chest imaging, which was more common in patients without influenza, and white blood cell count, which was higher among patients without influenza, PFIs were generally similar between patients with and without influenza (**Table 3**). The severity score was non-normally distributed among patients with influenza (**Fig 1**), with a median score of 4.5 (IQR, 2 to 8; range, 0–20), including 70 (33%) with a score ≥8 and 140 (67%) with a score <8.

**Table 3:** Comparison of Physiologic Features and Interventions (PFI)^1^ in hospitalized adults with lower respiratory tract infection, by influenza status.

	Influenza +		Influenza -		
	n = 210		n = 810		
	n	%	n	%	P-value
**Demographics**					
Age, years, Median (IQR)	63 (52–75)		62 (52–72)		0.34
Female	121	57.6	367	45.3	<0.01
**Vital Signs**					
Temperature, °C, Median (IQR)	37.4 (36.8–38.2)		37.0 (36.7–37.6)		<0.01
< 35.1	0	0	1	0.1	<0.01
35.1–36.0	4	1.9	22	2.7	
36.1–38.0	145	69.1	666	82.2	
38.1–39.0	32	15.2	92	11.4	
≥ 39.1	29	13.8	29	3.6	
Respiratory Rate, Breaths/Minute, Median (IQR)	21 (20–24)		20 (18–24)		0.26
<9	0	0	0	0	0.43
9–11	0	0	0	0	
12–20	103	49.1	438	54.1	
21–24	63	30.0	218	26.9	
>24	44	21.0	154	19.0	
Heart Rate, Beats/Minute, Median (IQR)	104 (91–116)		99 (87–112)		<0.01
≤40	0	0	0	0	0.05
41–59	5	2.4	26	3.2	
60–109	118	56.2	530	65.4	
110–130	69	32.9	201	24.8	
>130	18	8.6	53	6.5	
Mean Arterial Pressure, mmHg, Median (IQR)	91 (79–106)		94 (82–107)		0.03
< 60	9	4.3	18	2.2	0.1
≥60	201	95.7	792	97.8	
Oxygen Saturation, %	94 (90–97)		94 (90–96)		0.86
<90%	42	20	152	18.8	0.85
90–93%	58	27.6	238	29.4	
> 93%	110	52.4	420	51.8	
**Laboratory Values**					
WBC Count, x10^9^ Cells/L, Median (IQR) [missing = 1]	8.9 (6.2–12.0)		11.9 (8.6–16.1)		<0.01
<1	0	0	1	0.1	<0.01
1–2	10	4.8	6	0.7	
3–14	174	82.9	551	68.1	
15–19	15	7.1	155	19.2	
20–39	11	5.2	92	11.4	
≥40	0	0	4	0.5	
Sodium, mEq/L, Median (IQR)	137 (134–139)		138 (135–140)		0.05
<120	0	0	2	0.3	0.49
120–125	1	0.5	10	1.2	
>125	209	99.5	798	98.5	
Arterial Blood Gas, pH, Median (IQR) [missing = 550]	7.40 (7.36–7.44)		7.41 (7.38–7.45)		0.15
<7.25	4	4.6	11	2.9	0.62
7.25–7.32	6	7	34	8.9	
7.33–7.49	73	84.9	312	81.2	
7.5–7.59	3	3.5	25	6.5	
≥7.6	0	0	2	0.5	
**Outcomes and Interventions**					
Altered mental status	10	4.8	40	4.9	0.92
Infiltrate on Imaging (Chest Radiograph or CT Scan)	101	48.1	507	62.6	<0.01
Vasopressor Use	4	1.9	12	1.5	0.66
Noninvasive or Invasive Ventilation	14	6.7	53	6.6	0.26

Abbreviations: CT = computed tomography; IQR = interquartile range; WBC = white blood cell.

^1^ “Global” scale inclusive of clinical features, clinical interventions, laboratory values, and imaging findings.

Among patients with influenza, the severity score was influenced by radiographic findings of pulmonary infiltrate (48.1%), heart rate ≥110 beats/minute (41.4%), oxygen saturations of 90–93% (27.6%) or <90% (20.0%), respiratory rate >24 breaths/minute (21.0%), temperature ≥39.1°C or <36.0°C (15.7%), invasive or non-invasive mechanical ventilation (6.7%), white blood cell count >20,000/mm^3^ (5.2%), altered mentation (4.8%), and mean arterial pressure <60 mmHg (4.3%) [**Table 4**].

**Table 4:** Physiologic features and interventions among hospitalized influenza-positive patients by severity score.

Patients with Laboratory Confirmed Influenza	Severity Score < 8		Severity Score ≥ 8		
	n = 140		n = 70		
	n	%	n	%	P-value
**Demographics**					
Age, years, Median (IQR)	64.5 (55–77)		57.5 (45–69)		<0.01
Female	78	55.7	43	61.4	0.43
**Vital Signs**					
Temperature, °C, Median (IQR)	37.3 (36.8–37.9)		37.9 (37.1–39.1)		<0.01
< 35.1	0	0	0	0	<0.01
35.1–36.0	1	0.7	3	4.3	
36.1–38.0	112	80.0	33	47.1	
38.1–39.0	17	12.1	15	21.4	
≥ 39.1	10	7.1	19	27.1	
Respiratory Rate, Breaths/Minute, Median (IQR)	20 (18–22)		24 (20–28)		<0.01
<9	0	0	0	0	<0.01
9–11	0	0	0	0	
12–20	84	60.0	19	27.1	
21–24	45	32.1	18	25.7	
>24	11	7.9	33	47.1	
Heart Rate, Beats/Minute, Median (IQR)	99 (88–113)		114.5 (101–126)		<0.01
≤40	0	0	0	0	<0.01
41–59	4	2.9	1	1.4	
60–109	95	67.9	23	32.9	
110–130	34	24.3	35	50.0	
>130	7	5.0	11	15.7	
Mean Arterial Pressure, mmHg, Median (IQR)	91 (80–103)		92 (77–109)		0.69
< 60	2	1.4	7	10.0	<0.01
≥60	138	98.6	63	90.0	
Oxygen Saturation, %, Median (IQR)	94 (91–97)		92 (88–95)		<0.01
<90%	20	14.3	22	31.4	0.01
90–93%	40	28.6	18	25.7	
> 93%	80	57.1	30	42.9	
**Laboratory Values**					
WBC Count, x10^9^ Cells/L, Median (IQR)	7.7 (5.7–10.6)		11.2 (7.3–15.2)		<0.01
<1	0	0	0	0	<0.01
1–2	7	5.0	3	4.3	
3–14	126	90.0	48	68.6	
15–19	5	3.6	10	14.3	
20–39	2	1.4	9	12.9	
≥40	0	0	0	0	
Serum Sodium, mEq/L, Median (IQR)	137 (134–140)		136 (134–139)		0.24
<120	0	0	0	0	0.16
120–125	0	0	1	1.4	
>125	140	100	69	98.6	
ABG, pH, Median (IQR) [missing = 124]	7.41 (7.36–7.44)		7.40 (7.36–7.44)		0.84
<7.25	0	0	4	9.1	0.07
7.25–7.32	5	11.9	1	2.3	
7.33–7.49	36	85.7	37	84.1	
7.5–7.59	1	2.4	2	4.6	
≥7.6	0	0	0	0	
**Outcomes and interventions**					
Altered Mental Status	6	4.3	4	5.7	0.65
Infiltrate on Imaging (CXR or CT Scan)	38	27.1	63	90.0	<0.01
Vasopressor Use	1	0.7	3	4.3	0.07
Noninvasive or Invasive Ventilation	3	2.1	11	15.7	<0.01

Abbreviations: ABG = Arterial Blood Gas; CT = computed tomography; CXR = Chest Radiograph; IQR = interquartile range; WBC = White Blood Cell.

Patients with high severity scores (≥8) were younger (median age 57.5 years, IQR: 45 to 69 years) than those with lower scores (median age 64.5, IQR: 55–77). PFIs that distinguished low vs high severity with difference in prevalence of ≥20% included: pulmonary infiltrates (27.1% vs 90.0%), respiratory rate >24 breaths/minute (7.9% vs 47.1%), temperature ≥ 39.1°C or <36.0°C (7.1% vs 27.1%), and heart rate >110 beats/minute (29.3% vs 65.7%), and white blood cell count >15,000 (5.% vs 27.2%). In addition, as compared with patients with low severity, patients with high severity also were more likely to have oxygen saturations <90% (14.3% vs 31.4%) and to require invasive or non-invasive mechanical ventilation (2.1% vs 15.7%). Features that were uncommon or not different between high and low severity patients included serum sodium levels, altered mental status, vasopressor use, and arterial pH. No significant differences in underlying conditions were observed between those with severity score <8 or ≥8 (**Table 5**).

**Table 5:** Underlying conditions of hospitalized influenza-positive patients by severity score.

	Severity Score				
Patients with Laboratory Confirmed Influenza	Severity score < 8		Severity Score ≥ 8		
	n = 140		n = 70		
	n	%	n	%	P-value
**Underlying conditions**					
Active neoplasm	13	9.3	9	12.9	0.43
Congestive heart failure	32	22.9	14	20.0	0.64
Coronary artery disease	42	30.0	17	24.3	0.39
Atrial fibrillation	19	13.6	11	15.7	0.68
Hypertension	104	74.3	46	65.7	0.19
Hyperlipidemia	73	52.1	31	44.3	0.28
Stroke	18	12.9	8	11.4	0.77
Chronic renal disease	28	20.0	15	21.4	0.81
Liver disease	8	5.7	4	5.7	1.0
Diabetes mellitus	51	36.4	29	41.4	0.48
Chronic obstructive pulmonary disease	73	52.1	31	44.3	0.28
Home oxygen use	29	20.7	8	11.4	0.10
Hospitalization in past 90 days	25	17.9	16	22.9	0.39

## Discussion

Using a combination of severity scores to identify commonly available physiologic parameters of acute illness, we developed a quantitative scale that can grade illness severity of influeza-associated acute respiratory illness among hospitalized adults. Using a clinical trial dataset, we show that a gradient in severity exists among hospitalized patients with influenza and can be pragmatically quantified using vital signs, commonly available laboratory measurements, clinical findings, and critical interventions. In this cohort, highly prevalent findings that distinguished patients with higher scores from lower scores included pulmonary infiltrates, fever, and tachycardia; additional helpful features included respiratory rate and oxygen saturations. Such a scale could provide a standardized composite outcome for evaluating disease attenuating effects of influenza vaccination and improving reproducibility across studies. The IDEAS scale may have other applications as well, such as evaluating the clinical effectiveness of influenza antiviral therapies.

Studies evaluating the gradient of clinical severity of influenza virus infection in hospitalized patients from the perspective of immune-mediated disease attenuation or antiviral treatment are scarce. In experimental human virus challenge studies, pre-existing immunity or timely initiation of antiviral treatment can reduce symptom severity which correlates with viral replication and pro-inflammatory cytokine levels [2, 30–32, 35, 36, 38, 69, 70]. An absence of pre-existing immunity (e.g., through lack of vaccination) may result in progression of disease pathology from the upper respiratory tract to the lungs. Thus, measuring disease attenuating effects of vaccination or antiviral treatments would necessitate a standardized scale that differentiates acute disease limited to the upper airway from disease that has spread to the lower airway or systemically. However, published scales of disease severity do not differentiate these acute disease processes of influenza virus infection and are designed to predict outcomes such as length of stay, ICU admission, or mortality [54–58, 60, 61]. When evaluating attenuating effects of vaccination or antiviral treatments, use of such non-specific outcomes can lead to biases from other factors including physiologic reserve, healthcare seeking patterns, and admission practices. Thus, we extracted predictors of severity that we deemed to be less influenced by these biases, focusing on parameters common across studies and those consistent with acute lung injury or systemic manifestations of infection. In our cohort, we identified the upper quartile to have the greatest derangement in PFIs. We suspect that this upper quartile of patients on the IDEAS scale is a better representation of patients with severe pathophysiology related to cytokine storm, acute respiratory distress syndrome, or extrapulmonary involvement after influenza virus infection. Ideally, attenuation would be measured capturing the full spectrum of influenza from asymptomatic infection to severe illness. If vaccination attenuates disease among hospitalized patients, comparing vaccine effectiveness in the highest vs the lower quartile of the IDEAS scale could be a better representation of vaccination-mediated disease attenuation than using binary non-specific outcomes.

Typical features of lower respiratory tract involvement in previous influenza studies have included hypoxia and multi-lobar infiltrates, although fever, systemic manifestations of illness, and laboratory abnormalities are also common predictors of severity across studies [71–74]. We favored reduced missingness and improve practicality by focusing on factors that are routinely measured or easily accessible in clinical settings. For example, small single center studies have identified lactate and brain natriuretic peptide (BNP) as a predictor of poor outcomes in patients with community acquired pneumonia, but these were uncommonly measured markers in our dataset [75–77]. Interestingly, C-reactive protein has been a commonly measured marker and a reliable predictor of severity during the COVID-19 pandemic [78]. While, C-reactive protein levels have not been well-examined in patients with seasonal influenza, several small studies identified higher levels in patients with severe disease during the 2009 H1N1 pandemic [79]. Some studies during the 2009 H1N1 pandemic also identified predictors of influenza severity not included in our PFIs including IgM levels, age>45 years, sex, aspartate aminotransferase levels, or lactate dehydrogenase levels [72, 80, 81]. Use of PaO2/FiO2 or SpO2/FiO2 have also been favored over oxygen saturation alone in some studies, but FiO2 concentrations were not consistently recorded [62]. Scores grading the severity of pulmonary infiltrates on radiographs may be valuable rather than dichotomous interpretation, although this could require more specialized interpretation with potential issues of inter-rater reliability [73]. Our use of a broad range of PFIs likely has some collinearity with these variables that were not included in this study. Future refinement of these PFIs or adaptations to meet the needs of the database is encouraged. If a gradient is identified that reflects viral-mediated damage, the global severity scales should prove to be useful, reliable, and reproducible measure of evaluating the disease attenuating effects of vaccination or antiviral treatments.

We down-weighted pre-existing factors associated with derangements in PFIs unrelated to the acute infection that can misclassify patients as having severe LRTI. Here we considered age >70 years, COPD, and CHF to be such conditions because they are over-represented among patients hospitalized with LRTI and have baseline abnormalities that overlap with PFIs used to define severe influenza LRTI [44, 64, 66]. Other factors with similarly high prevalence may also warrant consideration when applying global IDEAS scale to other cohorts. For example, some cohorts may be derived from specialty hospitals with higher prevalence of immunocompromised patients. Trade-offs exist between parsimony and inclusiveness–not all pre-existing conditions or PFIs are practical choices to include in general severity score models. A falsely increased score due to a rare chronic condition or a lower score due to a missed PFI may misclassify disease severity for the individual patient, though this is less likely with an aggregate score using multiple PFIs, unless missingness is common across multiple PFIs. If misclassification occurs, it is most likely in patients with scores closer to the median value and thus approaches such as focusing on comparing effectiveness in the highest versus the lowest quartile could be used to evaluate disease attenuation.

Clinical studies evaluating disease attenuation associated with influenza vaccination have been scarce considering that vaccines were first developed over 80 years ago. Namely, most vaccine efficacy studies have focused on outcomes of any symptomatic influenza and not disease attenuation in vaccinated versus unvaccinated groups. A few clinical trials of inactivated and live-attenuated influenza vaccines in children have demonstrated higher efficacy against laboratory-confirmed influenza with severe endpoints (defined mostly by fever) compared with influenza of any severity [9, 82, 83]. Findings from observational studies in general have been inconsistent possibly due to differences in outcomes, sample size, age differences, and influenza season. Some studies have suggested disease is attenuated in association with vaccination, through indirect measures of severity, including decreased ICU admission and reduced length of hospital stay [8, 10, 42, 84–86]. A few studies have also demonstrated higher point estimates of vaccine effectiveness against critical influenza illness versus non-critical illness, with setting of care being a correlate of disease severity [10, 85]. In one outpatient study, influenza vaccination was associated with reduced perceived symptom severity across eight systemic, upper respiratory, and lower respiratory symptoms among older adults but not in younger adults [87]. Other studies have not found differences in vaccine effectiveness between inpatient and outpatient settings [43, 44], or reduction in risk of severe disease among vaccinated cases [88–90]. Vaccination-associated reduction in disease severity in some observational studies has also varied by age and season [8, 42, 87]. The development and use of a standardized ordinal scale that uses objective indicators and endpoints of severity such as IDEAS could be valuable for evaluations of attenuation mediated by vaccination and antiviral treatment. Such scales have been successfully used for pertussis, varicella, and rotavirus disease to demonstrate vaccine effectiveness against progression of disease (termed VEp), conditioned on infection, as well as overall vaccine effectiveness of reducing susceptibility to a given outcome in the general population (termed VEs) [11, 13, 91]. Such analyses are key for evaluating whether partially protective vaccines can limit the severity of breakthrough infections in vaccinated persons as compared with infections in unvaccinated persons.

Our study should be interpreted in the context of some limitations. First, the IDEAS scale applied physiologic features and interventions that were based largely on studies evaluating all-cause pneumonia and sepsis, which are well-studied, and thus may be less specific to influenza that might also be affected by prior vaccination or infection history. Second, the selection of the PFIs was also informed by experimental inoculation studies in animals and humans and understanding of mechanisms of immunity as well as antiviral treatments [2, 30–32, 35, 36, 38, 69, 70]. Thus, the assumption that these features of influenza severity are relevant to measuring attenuating effects of vaccination or antiviral treatments cannot be empirically validated. Third, misclassification of patients into varying gradients of severity is possible if some of the included PFIs were affected for reasons unrelated to influenza virus replication or were missing non-randomly. However, the aggregated score based on multiple PFIs reduces misclassification related to derangements in individual PFIs. Fourth, the cut-offs for quantitative PFIs may not precisely correlate with severe disease in individual patients but have predicted severe outcomes in prior studies and were generally consistent across studies. Small alterations in cutoffs of the features are less likely to influence the severity grading when applying an aggregated score in a cohort of patients. Lastly, the generalizability of the score may vary but the score can be adapted or modified to identify a gradient of severity within a given dataset. The IDEAS scale was based on hospitalized patients alone and would not be relevant to outpatient settings but could be applied to grade severity in a large cohort that captures the breadth of outpatient and inpatient visits.

In summary, we reviewed outcomes-based sepsis, pneumonia, and influenza severity scores to identify objective physiologic features and interventions that differentiate a breadth of severity observed in patients hospitalized with influenza. Under the premise that vaccination and antiviral treatment reduces disease severity, reductions in the score based on these features would be expected in vaccinated patients or those receiving timely antiviral treatment. Further adaptation and implementation of the IDEAS scale under real-world settings might improve reproducibility and validity of trials and studies evaluating influenza disease attenuation after vaccination or antiviral treatment.

## Supporting information

S1 Data (XLSX)
